# Genome-wide association study identified six loci associated with adverse drug reactions to aripiprazole in schizophrenia patients

**DOI:** 10.1038/s41537-023-00369-6

**Published:** 2023-07-25

**Authors:** Xueping Wang, Dongli Mei, Zhe Lu, Yuyanan Zhang, Yaoyao Sun, Tianlan Lu, Hao Yan, Weihua Yue

**Affiliations:** 1 Peking University Sixth Hospital, Peking University Institute of Mental Health, Beijing, China; 2 National Clinical Research Center for Mental Disorders & NHC Key Laboratory of Mental Health (Peking University), 100191 Beijing, China; 3 Research Unit of Diagnosis and Treatment of Mood Cognitive Disorder, Chinese Academy of Medical Sciences (No. 2018RU006), Beijing, China; 4 School of Nursing, Peking University, 10019 Beijing, China; 5 PKU-IDG/McGovern Institute for Brain Research, Peking University, Beijing, China

**Keywords:** Schizophrenia, Biomarkers

## Abstract

Aripiprazole is recommended for routine use in schizophrenia patients. However, the biological mechanism for the adverse drug reactions (ADRs) among schizophrenia patients with the antipsychotic drug aripiprazole is far from clear. To explore the potential genetic factors that may cause movement-related adverse antipsychotic effects in patients, we conducted an association analysis between movement-related ADRs and SNPs in schizophrenia patients receiving aripiprazole monotherapy. In this study, multiple ADRs of 384 patients were quantified within 6-week treatment, and the scores of movement-related ADRs at baseline and follow-up time points during treatment were obtained. The highest score record was used as the quantitative index in analysis, and genetic analysis at the genome-wide level was conducted. The SNP rs4149181 in *SLC22A8* [*P* = 2.28 × 10^−8^] showed genome-wide significance, and rs2284223 in *ADCYAP1R1* [*P* = 9.76 × 10^−8^], rs73258503 in *KCNIP4* [*P* = 1.39 × 10^−7^], rs678428 in *SMAD9* [*P* = 4.70 × 10^−7^], rs6421034 in *NAP1L4* [*P* = 6.80 × 10^−7^], and rs1394796 in *ERBB4* [*P* = 8.60 × 10^−7^] were found to be significantly associated with movement-related ADRs. The combined prediction model of these six loci showed acceptable performance in predicting adverse events [area under the curve (AUC): 0.84]. Combined with the function and network of the above genes and other candidate loci (*KCNA1*, *CACNG1*, etc.), we hypothesize that *SLC22A8* and *KCNIP4*-Kv channel perform their respective functions as transporter or channel and participate in the in vivo metabolism or effects of aripiprazole. The above results imply the important function of ion transporters and channels in movement-related adverse antipsychotic effects in aripiprazole monotherapy schizophrenia patients.

## Introduction

Schizophrenia is a serious mental disorder with high inheritability^1,2^. It mainly manifests in clinical emotion, thinking, cognition, behavior and social functions. Epidemiological investigation shows that the prevalence rate of schizophrenia in the general population is approximately 1%^1^, and it usually starts slowly or onset at a young age and lasts for one’s lifetime. The process of schizophrenia is prolonged and repeated, which can lead to mental disability and bring a heavy burden to patients, family members and society. Due to the heterogeneity of the disease, the exact pathological mechanism of schizophrenia has not been clearly elucidated. It is generally believed that both genetic susceptibility and environmental factors play roles in the occurrence and development of the disease. Antipsychotic drugs can relieve patients’ clinical symptoms, but approximately 75% of patients have given up treatment due to poor efficacy or side effects of drugs^3^. Common adverse drug reactions to antipsychotics include extrapyramidal effects, headache, weight gain, and QTc prolongation^4^. Environmental factors, biological factors and therapeutic strategies may be the fickle factors behind the difference in adverse drug effects^5^. Serious adverse effects bring inconvenience and psychological pain to patients. Studying pharmacogenetics and identifying the genetic factors within adverse drug reactions can help us develop personalized medication guidance and cope with serious adverse symptoms. Among different adverse drug reactions, the symptoms derived from the motor and nervous systems are particularly complex. Therefore, a clear and standardized monotherapy design can help us analyze the underlying mechanisms of movement-related adverse reactions.

Compared with first-generation antipsychotics, atypical/second-generation antipsychotics have fewer extrapyramidal and asthenia-related adverse reactions. Aripiprazole is an atypical antipsychotic and is also known as a third-generation antipsychotic^6,7^. The pharmacological mechanism of aripiprazole is mainly due to partial agonist activity at D_2_ and 5-HT_1A_ receptors and the potent antagonism of the 5HT_2A_ receptor^8,9^. Its metabolism is mainly through the dehydrogenation and hydroxylation of CYP3A4 and CYP2D6 enzymes^10^. The possible adverse reactions caused by aripiprazole include headache, insomnia, dizziness, and restlessness^4,11^. Aripiprazole has less effect on weight change^4^. Compared with olanzapine and risperidone, aripiprazole is less likely to cause metabolic adverse drug reactions^4^. However, genome-wide pharmacogenomics studies on the adverse drug reactions of aripiprazole are very limited. Previous studies mainly focused on the pharmacodynamics of *DRD2* and *5-HTR2A* and the response to negative symptoms and cognitive performance^12–15^. Movement-related adverse events, including extrapyramidal side effects, motor restlessness and other abnormal movements, show individual differences. The genetic basis underlying the individual difference needs to be discovered.

Investigating the genetic mechanism of the movement-related adverse drug response to aripiprazole is an urgent research direction. Therefore, we conducted a genome-wide association analysis in schizophrenia patients treated with aripiprazole monotherapy to explore the genetic loci related to the severity of movement-related antipsychotic effects.

## Subjects and methods

### Study design and participants

A total of 431 patients were recruited in the discovery cohort study. According to the study protocol, we performed routine baseline assessments at the start, including the general information records, the DSM-IV-TR, and the inclusion/exclusion criteria selection. Within 2 weeks after clinical inclusion, clinicians adjusted the aripiprazole dosages based on the treatment effectiveness (10–30 mg/day). After that, the dosages remained unchanged throughout the study period. The patients received clinical evaluation at weeks 2, 4, and 6, and the assessments of adverse drug reactions were recorded. Blood samples were collected at baseline. All the clinicians and patients were blinded in the study design. The patient could decide to leave the study at any time, and then the patient will be dropped from the study. In some cases, the patient cannot continue to participate, or the clinician cannot contact the patient. If a patient did not complete the full follow-up study, the last-observation carried-forward procedure was applied, and the last recording was represented as his/her treatment response. Combined with the SNP detection and quality control results, 384 patients were included in the information and polymorphism detection results in the analysis stage. The study was approved by the research ethics committees of hospital.

The inclusion conditions of the subjects in this study were as follows: (1) diagnosed with schizophrenia based on the Structured Clinical Interview of the *Diagnostic and Statistical Manual of Mental Disorders*, fourth edition, Text Revision (DSM-IV-TR); (2) aged 18–45 years; (3) Han Chinese lineage; (4) total scores more than 60 on the Positive and Negative Syndrome Scale (PANSS); and (5) provided written informed consent. The exclusion criteria were as follows: (1) pregnancy or breast-feeding; (2) malignant syndrome or acute dystonia, well-documented histories of epilepsy and hyperpyretic convulsion; (3) a DSM-IV diagnosis of alcohol or drug dependence, or a history of drug-induced neuroleptic malignant syndrome; (4) had previously attempted suicide, or had experienced the symptoms of severe excitement and agitation; (5) severe or unstable physical diseases, such as abnormal liver or renal function; (6) requirement of long-acting injectable medication to maintain treatment adherence or regularly treated with clozapine for treatment over the past month; (7) had QTc prolongation, a history of congenital QTc prolongation within the past 6 months. The validation samples were from the Chinese Antipsychotics Pharmacogenetics Consortium (CAPEC), and the research protocol had been documented in the previous article^16^. In this set of data, serious adverse reactions such as akathisia were recorded whether they occurred at multiple timepoint. We extracted the SNPs genotype of five significant candidate gene (rs2284223 undetected in current genomic data). We used the gene’s overall risk score to predict whether patients had an adverse effect of akathisia.

### Phenotype definition

In discovery cohort study, the adverse drug reaction scores for movement-related antipsychotic effects are the sum of three assessment scales, including the Barnes Akathisia Rating Scale (BARS), the Abnormal Involuntary Movement Scale (AIMS), and the Simpson-Angus Scale (SAS). At baseline, all patients were evaluated with the above assessment scales, and their psychiatric symptoms and adverse drug reactions were evaluated by clinicians at 2, 4, and 6 weeks of follow-up. The BARS is scored according to its instructions^17^. Objective akathisia, subjective awareness of restlessness, and subjective distress related to restlessness are rated on a 4-point scale, and the score ranges from 0 to 3. The global clinical assessment of akathisia uses a 5-point scale ranging from 0 to 4. Therefore, the summed total score ranges from 0 to 9. The AIMS is a scale designed to assess abnormal involuntary movement^18^, primarily tardive dyskinesia. This scale includes 12 items, and items 1–10 are graded from 0 to 4 (except items 11 and 12). The SAS is a rating scale used to assess extrapyramidal side effects with 10 items ranging from 0–4^19^. The adverse drug reaction phenotype of validation sample was recoded as text tag, such as akathisia, insomnia, tachycardia, etc.

### Genotyping

Genomic DNA was extracted using the QIAamp DNA Mini Kit (QIAGEN, Hilden, Germany). The samples were genotyped with Human OmniZhongHua-8 Beadchips (Illumina, San Diego, CA, USA, http://www.illumina.com/products/human-omni-zhonghua.html), which were specifically designed for the Chinese population genome as a gene detection chip. Preliminary quality control of genome data was performed before the association analysis. Sample results in the following conditions were discarded: (1) the genotype call rate was less than 98%, (2) in the case of gender discordance, (3) samples from the individuals were first-degree or second-degree relatives, (4) samples were genetic outliers, (5) SNP minor allele frequency was less than 0.05, and (6) *P*-values for Hardy–Weinberg equilibrium were less than 1 × 10^−6^. Genotype imputation for the samples was performed with the prephasing imputation stepwise approach performed in IMPUTE2 and SHAPEIT. Haplotypes derived from phase I of the 1000 Genomes Project (release version 3) were used as references.

### Statistical analyses

We hypothesized that adverse drug reactions in patients treated with aripiprazole were associated with their own genotypes and conducted association analyses at the genome-wide level. After quality control, linear regression under an additive genetic model was implemented to evaluate the associations between allele dosage and adverse drug reaction scores in PLINK (version 1.90)^20,21^. Gender, age, baseline adverse effect score, medication dose, and the first five principal components of population structure were used as covariates in our analysis. After that, we used a *P*-value less than 5 × 10^−8^ as the data threshold for genome-wide significance. To explore more adjacent significant sites, significance levels less than 1 × 10^−6^ and 1 × 10^−5^ were also analyzed. We used the R (version 4.2.2) CMplot package to draw the Manhattan plot. Receiver operating characteristic (ROC) analyses were performed using GraphPad Prism 6. Gene Ontology (GO) and pathway enrichment analyses for candidate genes identified by genome-wide association were performed with the R package clusterProfiler. The backup data are from the Database for Annotation, Visualization and Integrated Discovery (DAVID, https://david.ncifcrf.gov/).

## Results

### ADRs scores indicate the movement-related adverse antipsychotic effects

In this study, we recruited a total of 431 schizophrenia patients, and 384 patients finally passed the clinical and genotype data quality control. The movement-related adverse response was represented by the ADRs score, which was obtained by summing the scores of the three scales, including the Barnes Akathisia Rating Scale (BARS), the Abnormal Involuntary Movement Scale (AIMS), and the Simpson-Angus Scale (SAS). Table 1 shows the age, sex ratio, average aripiprazole dose and ADRs Score. The maximum quantitative value of ADRs scores was used for analysis, and the baseline value was subtracted to eliminate the baseline effect.

**Table 1:** Demographic and clinical characteristics of 384 patients following 6 weeks of aripiprazole monotherapy.

Index	Mean ± SD or *n* (%)
**Age at study entry, years**	31.1 ± 7.8
**Gender,** ***n*** **(%)**	
Men/women	182 (47.4)/202 (52.6)
**Doses of medication, mg**	23.9 ± 6.10
**ADRs scores**^a^	4.07 ± 5.18

^a^ADRs scores are the sum from three assessment scales, including the Barnes Akathisia Rating Scale (BARS), the Abnormal Involuntary Movement Scale (AIMS), and the Simpson-Angus Scale (SAS).

### Genome-wide association results of aripiprazole treatment movement-related ADRs

**Fig. 1:**
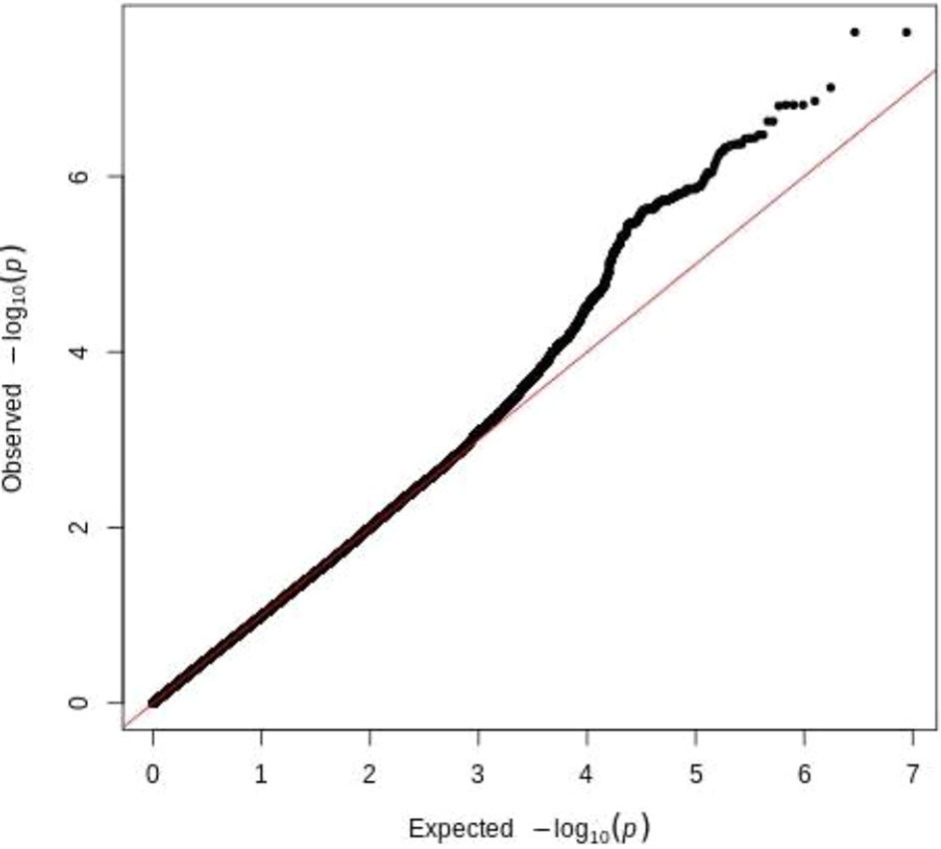
Quantile–quantile for the ADRs sample. The plot was generated with the data after quality control in PLINK and R software.

**Fig. 2:**
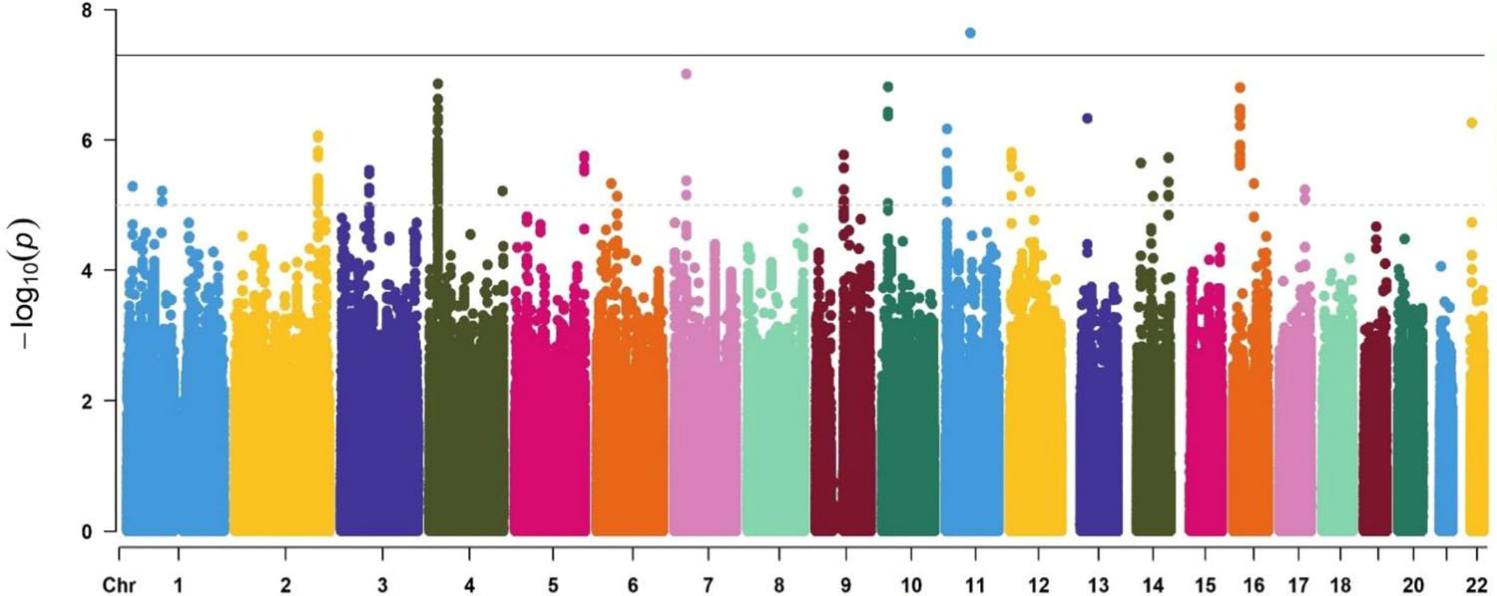
Manhattan plots for the ADRs sample. Genome-wide *P*-values [-log_10_ (p); *y*-axis] of SNPs were plotted against their physical positions on chromosomes (*x-*axis). The black line shows the genome-wide significance level (5 × 10^−8^); the dashed line shows the significance level for 1 × 10^−5^.

To identify genetic loci that might influence ADRs in aripiprazole treatment, genome-wide association analysis was performed. Figure 1 shows the quantile‒quantile plots, and Fig. 2 is the Manhattan plot for ADRs samples. Here, 4386312 SNPs were analyzed for movement-related adverse effects, and the linear regression analysis model was used in PLINK. Table 2 shows the 6 genes that reached (*P* < 1 × 10^−6^) significance. The genetic locus rs4149181 in *SLC22A8* shows genome-wide significance [*P* = 2.28 × 10^−8^], and rs2284223 in *ADCYAP1R1*, rs73258503 in *KCNIP4*, rs678428 in *SMAD9*, rs6421034 in *NAP1L4*, and rs1394796 in *ERBB4* show significance with *P* < 1 × 10^−5^. Among the 6 genes, the SNPs located in the *KCNIP4* gene intron showed the best continuity in the Manhattan plot (Fig. 2, Supplementary Fig. S1). There were 120 SNPs located in *KCNIP4* reaching the significance threshold (*P* < 1 × 10^−5^). The *KCNIP4* gene is highly expressed in brain tissues and mainly plays a role in synaptic function. The other 5 genes had different spatiotemporal expression patterns in different brain regions (Supplementary Figs. S2–S5). Moreover, the SNPs of *ADCYAP1R*, *KCNIP4*, *SMAD9*, *NAP1L4*, and *ERBB4* had eQTL effects on themselves in the brain (Supplementary Figs. S6 and S7). There are four gene loci (rs146319527, rs4747269, rs238842, and rs9605090) that are significantly associated with ADRs, but these loci currently lack gene annotation including RP11-17E2.2, 26 kb 3′ of RP11-461K13.1, CTA-481E9.4, and 14 kb 3′ of *RTN4R* (Supplementary Table S1).

**Table 2:** Genome-wide association results of aripiprazole treatment ADRs.

CHR	SNP	Position	Minor allele	Major allele	Frequency of minor allele	Functional annotation	Gene	BETA	SE	*P*-value
11	rs4149181	62781921	G	A	0.05339	intronic	*SLC22A8*	4.436	0.7767	2.28 × 10^−8^
7	rs2284223	31111441	C	T	0.2947	intronic	*ADCYAP1R1*	2.166	0.3982	9.76 × 10^−8^
4	rs73258503	21872346	C	T	0.1772	intronic	*KCNIP4*	2.506	0.4666	1.39 × 10^−7^
13	rs678428	37484026	G	A	0.05469	intronic	*SMAD9*	4.077	0.7949	4.70 × 10^−7^
11	rs6421034	3007647	C	T	0.122	intronic	*NAP1L4*	2.769	0.5478	6.80 × 10^−7^
2	rs1394796	213270427	T	C	0.06397	intronic	*ERBB4*	3.903	0.7796	8.60 × 10^−7^

### Potential predictive effect of gene loci on movement-related ADRs

**Fig. 3:**
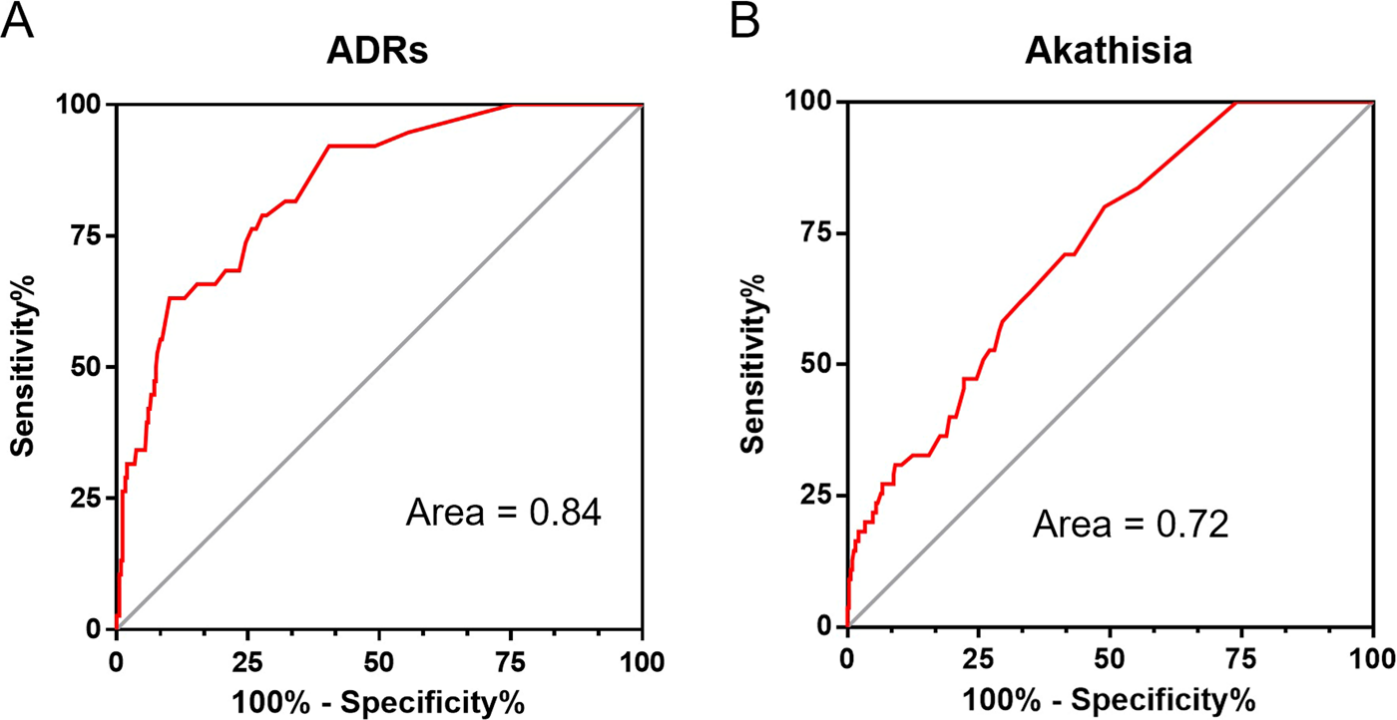
Potential predictive effect of the six SNPs and ADRs scores. **A** ROC analysis indicated that the six most significant SNPs could effectively predict the occurrence of serious adverse reactions (ADRs), and the threshold line for serious movement-related adverse reactions in this analysis was defined as 10 or above. **B** The predictive effect of six SNPs in the occurrence of akathisia during treatment.

Since the six candidate genes were all significant (*P* < 1 × 10^−6^), we tried to use them to predict the movement-related adverse effects of aripiprazole. The different ADRs threshold values were applied to the receiver operating characteristic curve (ROC curve) analysis, and Fig. 3A shows the predictive effect of six SNPs to distinguish movement-related adverse responses. In the classification model, patients with ADRs scores greater than 10 points (including 10 points) were considered to have relatively serious movement-related adverse reactions. The different alleles of SNPs were weighted by the coefficient factor of the linear regression. The area under the curve (AUC) was 0.84, with 38 serious adverse response patients. Akathisia is usually seen as a more problematic adverse reaction. The six SNPs also showed a valuable potential predictive effect (AUC = 0.72), which was derived from 54 patients who had akathisia during treatment (Fig. 3B). We tested the predictive effect of our candidate risk genes in a small sample. Among 39 patients who met the pharmacogenomic analysis requirements, adverse drug reaction risk scores were calculated for five SNPs (Supplementary Fig. S8). In the clinical records of adverse drug reactions, there were four patients with severe akathisia. Compared with the ranking of risk scores, the cases in the top three of the ADRs score all had severe akathisia. This further verifies the predictive effect of our analysis results.

**Fig. 4:**
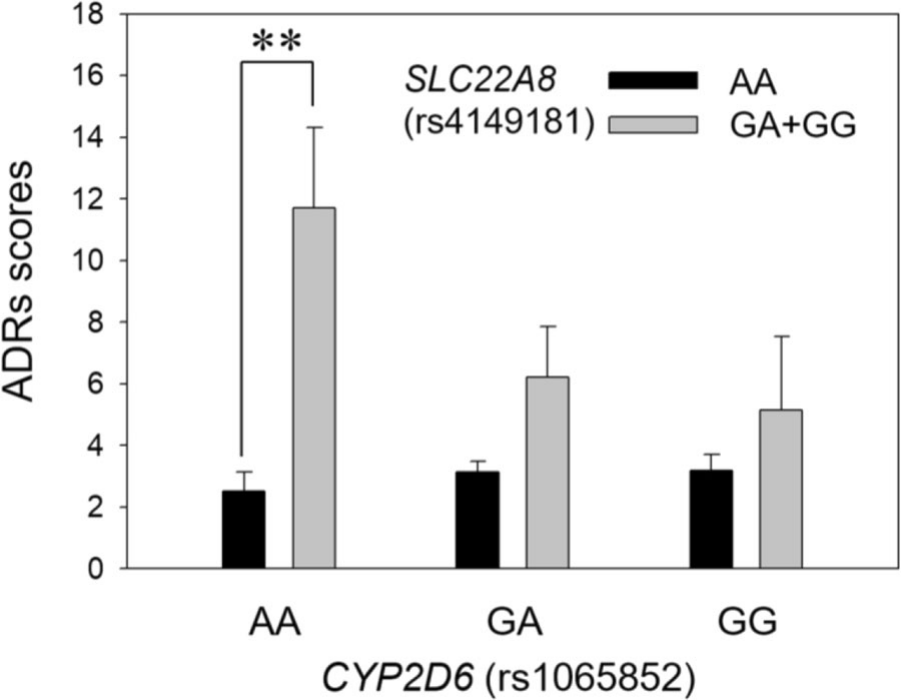
The ADRs of *SLC22A8* affected by *CYP2D6*. The ADRs scores of SNP (rs4149181) alleles in *SLC22A8* affected by CYP2D6*10 (rs1065852). Box plot of rs4149181 (AA/GA + GG) and the ADRs scores depending on *CYP2D6* polymorphism. The Kruskal‒Wallis test was used for comparisons between groups with Bonferroni correction. In the CYP2D6*10 AA genotype background, the alleles of rs4149181 were significantly different (**P = 0.00143, data are shown as the mean ± SE; AA, 2.53 ± 0.61; GA + GG, 11.71 ± 2.60).

Aripiprazole is mainly metabolized by CYP2D6 and CYP3A4. As *SLC22A8* has the highest expression in the kidney, we explored the possible function of *SLC22A8* in drug metabolism. *CPY2D6* genotyping has been suggested in personalized aripiprazole dosing^10^, and CYD2D6*10 has been characterized as a significantly decreased function allele^22^. Here, we extracted the genotype of CYD2D6*10 (rs1065852) and analyzed the adverse drug reaction score depending on the genotypes of CYD2D6*10 and *SLC22A8*. The G allele of rs4149181 is the higher-risk allele of *SLC22A8*. In the decreased function allele of CYP2D6*10 (Fig. 4), the risk genotype carriers showed higher adverse reactions, and the difference was significant (*P* = 0.00143; AA, 2.53 ± 0.61, *n* = 91; GA + GG, 11.71 ± 2.60, *n* = 7).

### GO and pathway analysis of genome-wide association results

**Fig. 5:**
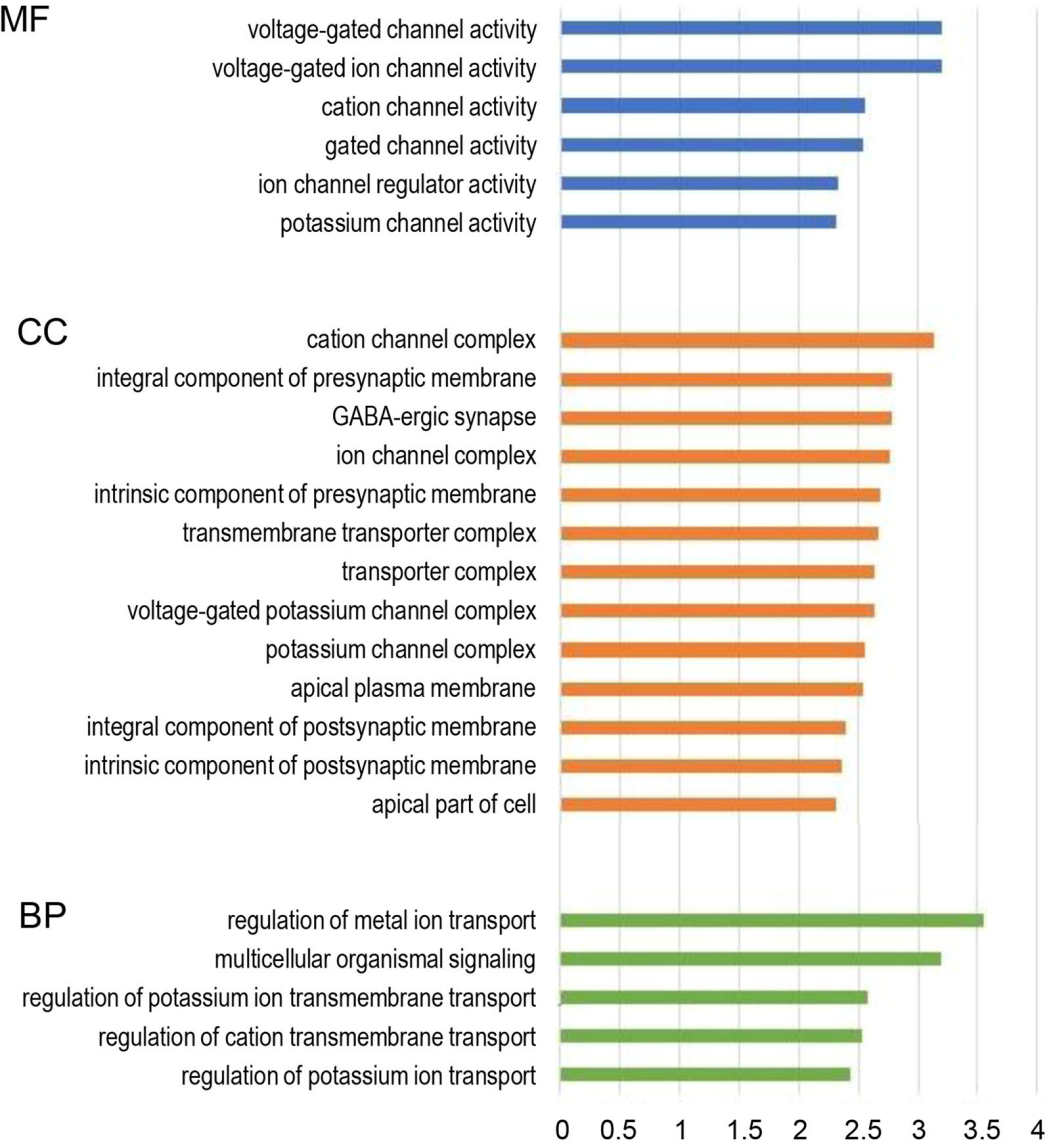
GO terms identified by genome-wide association results of movement-related adverse antipsychotic effects. MF molecular function, CC cellular component, BP biological process. Data are shown with *P* < 0.005.

To discover the molecular function and cellular pathway involved in the movement-related adverse effects of aripiprazole, GO and pathway enrichment analyses were applied with the candidate genes with a significance level less than 1 × 10^−5^. From gene annotation, there are several genes related to ion transporters or ion channels, including *SLC22A8*, *KCNIP4*, *KCNA1* and *CACNG1* (Table 2 and Supplementary Table S2). Figure 5 shows the GO terms, and detailed data on the GO terms are shown in Supplementary Table S3. Consistent with the gene function, channel or transporter functions, including voltage-gated ion channel activity (*P* = 0.000615) and regulation of metal ion transport (*P* = 0.000285), are closely related to movement-related adverse reactions induced by aripiprazole.

Previous research has reported that aripiprazole could inhibit Kv1.4 and Kv4.3 channel opening in a concentration-dependent manner^23^. In this study, we found a strong association between the *KCNIP4* gene and adverse reactions during aripiprazole treatment in schizophrenia patients, while *KCNA1*/*Kv1.1* also showed a significant association (rs57468930, *P* = 1.56 × 10^−6^). As *KCNIP4* and *KCNA1*/*Kv1.1* are both dominantly expressed in the brain, the protein‒protein interaction was further tested using a database (PPI website, https://string-db.org/). As shown in Supplementary Fig. S9, the interaction between KCNIP4 and KCNA1 had medium to high confidence (interaction score = 0.503).

## Discussion

In this study, we explored the possible genetic loci and susceptible functional genes associated with movement-related adverse reactions to aripiprazole monotherapy in schizophrenia patients. The rs4149181 in *SLC22A8* [*P* = 2.28 × 10^−8^] reached genome-wide significant associations with ADRs. The rs2284223 in *ADCYAP1R1*, rs73258503 in *KCNIP4*, rs678428 in *SMAD9*, rs6421034 in *NAP1L4*, and rs1394796 in *ERBB4* showed significant associations [*P* < 1 × 10^−6^].

*SLC22A8*, also known as *OAT3*, is mainly expressed in the kidney and is also expressed in the brain, retina, and testis^24^. It belongs to the SLC22 family that encodes organic anion transporters, and this gene family participates in drug absorption, disposition, and/or excretion^25^. The substrates of SLC22A8 include anionic drugs, estrone sulfate, bile acids, flavonoids, etc. Evidence from gene knockout animals confirmed the function of SLC22 transporters in pharmacological and toxicological effects^24^. The genetic heterogeneity of the SLC22 family affects transporter activity^26^, such as rs45566039 (p.R149C), resulting in reduced transport capacity^27^. Through the combined analysis of the two genotypes, we hypothesized the presence of *CYP2D6* and *SLC22A8* risk genes might play a synergistic role in drug metabolism and clearance in vivo. The above drug metabolism and clearing are carried out in the liver and kidney, respectively. When the functions of both organs are affected, more serious adverse reactions will be produced.

*KCNIP4* is a member of the Kv channel-interacting protein family, and it has a conserved EF-hand-like calcium-binding motif in the C-terminus^28,29^. The *KCNIP4* gene is mainly expressed in the brain and plays a role in neurodevelopment and neurite outgrowth^30^. Previous studies have shown that two SNPs (rs876477, *P* = 2.69 × 10^−5^; rs16871892, *P* = 0.0109) of *KCNIP4* were correlated with attention-deficit/hyperactivity disorder (ADHD) in children and adults^31,32^. In the genome-wide association results of schizophrenia in the CATIE study, rs1380272 (OR = 0.0522, *P* = 1.10 × 10^−5^) is an intron variant in the *KCNIP4* gene locus^33^. In an association screen analysis of chromosome 4 for three major psychiatric disorders, including schizophrenia, bipolar and major depressive disorder, researchers identified *KCNIP4* as the outstanding gene that might build a logical relationship among these disorders^34^. *KCNIP4* is significantly associated with suicidal ideation in antidepressant treatment-related suicidal ideation^35^, and it also serves as a cell-type-specific module in Autism’s Pathogenesis^36^. In a genome-wide association study of ACE inhibitor-induced cough, *KCNIP4* was significantly associated (OR = 1.3, *P* = 1.0 × 10^−8^) with ACEi-induced cough risk^37^. The *KCNIP4* gene was originally cloned as a binding partner of Presenilin 2 (PS2), and it can co-form a complex with the voltage-gated A-type K^+^ channel Kv4.2 and modulate its function in the brain^28,38^. In a study of glutamate-induced excitotoxicity, excessive expression of *KCNIP4* can produce protective effects on toxic nerves^39^. In conclusion, the *KCNIP4* gene is deeply involved in normal brain function activities, and its gene polymorphism is generally associated with mental disorders. Moreover, the protein‒protein interaction clues between KCNIP4 and KCNA1 are highly consistent with their function in the central nervous system, as KCNA1 was identified as a pathogenic gene for epileptic ataxia and dyskinesia^40,41^. Combined with the evidence of the channel open blockade effect of aripiprazole on Kv channels, we hypothesized that there might be a Ca^2+^ signal-KCNIPs-Kv pathway involved in the movement-related adverse response in aripiprazole treatment.

Other candidate genes, including *ADCYAP1R1*, *NAP1L4*, and *ERBB4*, have been reported in mental disorders. The *ADCYAP1R1* gene encodes a type I adenylate cyclase-activating polypeptide receptor and is highly expressed in the brain. Most clinical studies of the *ADCYAP1R1* gene have focused on post-traumatic stress symptoms and children’s fear conditioning^42–46^. The results of a meta-analysis showed that the C allele of rs2267735 may increase the risk of PTSD, and the risk effect was higher in women^47^. Moreover, rs2267735 is associated with major depression symptoms in trauma-exposed women^48^, and women with lower serum estradiol and lower *ADCYAP1R1* expression showed higher PTSD symptoms^44^. *ERBB4* encodes a receptor tyrosine kinase and promotes inhibitory synapse formation in pyramidal neurons^49^. ERBB4 mediates amyloid β-induced neurotoxicity, which is a biomarker for Alzheimer’s disease^50^, and Neuregulin1-ERBB4 signaling regulates the inflammatory pain of electroacupuncture analgesia in the spinal cord^51^. Moreover, the function of ERBB4 in dopamine neurons is related to depression-like behaviors, and it regulates the homeostasis of extracellular dopamine and norepinephrine in catecholaminergic cells^52^. In animal models, *ErbB4* (the homologous gene in mouse) has been shown to work with *NRG1* to maintain glutaminergic activity in the amygdala, and *ErbB4* is sufficient and crucial for tone-cued fear conditioning^53^. Loss of *ErbB4* leads to dendritic spine loss in excitatory neurons, and the dendritic spine loss also occurs in many psychiatric disorders^54^. *NAP1L4* is widely expressed in neurons and glial cells. It was reported that NAP1L4 interacts with DGKζ to attenuate hypoxic stress in the brain^55^. *SMAD9* is involved in bone morphogenetic protein (BMP) signaling and is associated with high bone mass^56^. It acts as a transcriptional regulator in BMP signaling^57^. Its polymorphism was associated with the risk of essential hypertension in the Chinese population^58^.

In this study, a genome-wide association analysis was conducted for movement-related adverse antipsychotic reactions in patients treated with aripiprazole monotherapy, and candidate genes such as *SLC22A8* and *KCNIP4* were identified. Previous studies analyzing movement-related adverse effects in schizophrenic patients with unrestricted drug use or using different omics datasets for secondary analysis have identified some susceptible gene loci^59,60^. This study provides new pharmacogenomic evidence and potential signaling pathways for aripiprazole treatment and constructs an adverse reaction prediction model. Since the three rating scales (SAS, BARS, and AIMS) are also used in the clinical assessment of tardive dyskinesia (TD), our results might also have important value in TD prediction. From our perspective, the main limitation of this study is the sample size of validation cohort, and further cross-validation and calculation of genetic risk prediction models in other cohorts or disease groups.

In conclusion, we elucidated the role of ion transporter genes and their associated regulatory proteins in movement-related antipsychotic effects in aripiprazole treatment in schizophrenia patients by genetic association analyses. The important functional genes found in this study, such as *SLC22A8*, *ADCYAP1R*, and *KCNIP4*, will be important candidates for further research on the molecular signaling pathways of mental and nervous system diseases. These genes may become specific drug targets for future treatment of difficult clinical problems such as akathisia. The *KCNIP4* was significantly associated with ADHD, autism, schizophrenia, bipolar and major depressive disorder, and this also suggested that K^+^ channel-related calcium regulator protein may be a common genetic basis in various mental disorders.

## Supplementary information

Supplemental materials -- Revised clean version

## Data Availability

The data of our study has been submitted to the Population Health Data Archive of National Population Health Data Center (NPHDC, https://www.ncmi.cn). The dataset’s accession number is 2016YFC1307000.
