# Supplementary material for: Genome-wide association study identified six loci associated with adverse drug reactions to aripiprazole in schizophrenia patients
Source: Schizophrenia (Heidelb). 2023 Jul 25;9(1):44. doi: 10.1038/s41537-023-00369-6 (PMC10368716; doi:10.1038/s41537-023-00369-6)
Supplement: Supplementary file 1 — Supplemental materials -- Revised clean version [file 41537_2023_369_MOESM1_ESM.pdf]

## SUPPLEMENTARY FIGURES

Figure S1. Regional plot of the loci associated with movement-related adverse antipsychotic effects at KCNIP4. The regional plot of genetic association study was performed on the LocusZoom (http://csg.sph.umich.edu/locuszoom/).

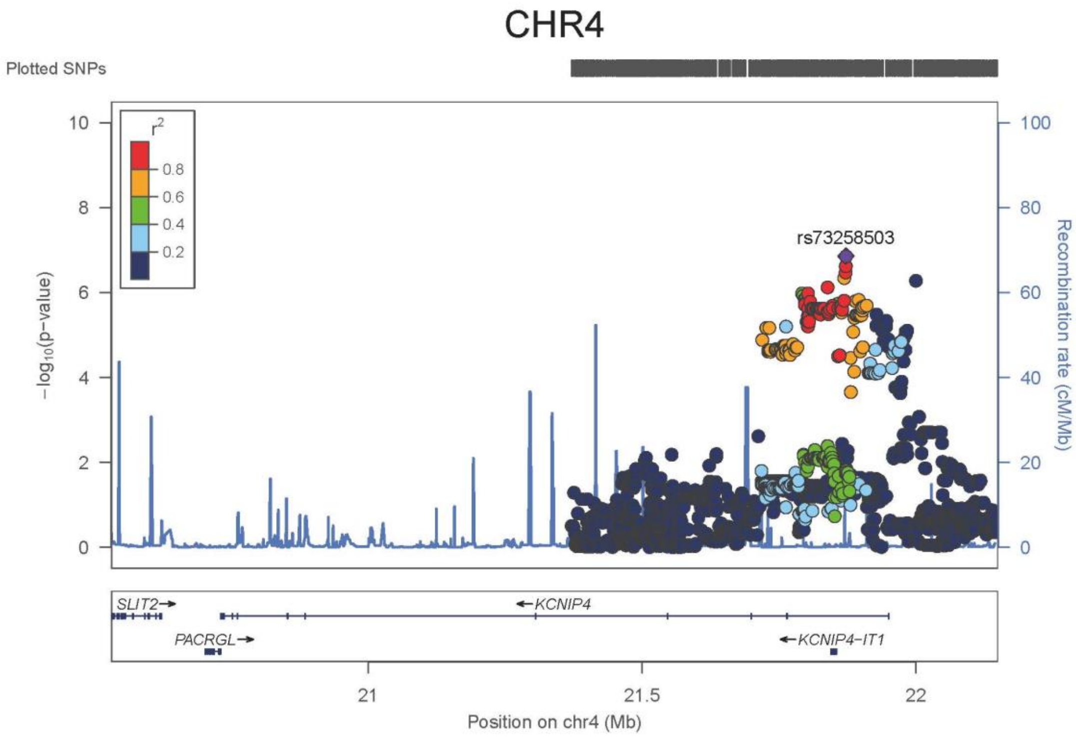

Figure S2. The spatiotemporal expression pattern of *SLC22A8*, *ADCYAP1R*, *KCNIP4* in human brain tissues. The dynamic gene expression along entire development and adulthood in the cerebellar cortex (CBC), mediodorsal nucleus of the thalamus (MD), striatum (STR), amygdala (AMY), hippocampus (HIP) and 11 areas of neocortex (NCX). The spatiotemporal expression patterns of candidate genes were analysed on Human Brain Transcriptome (HBT, https://hbatlas.org/).

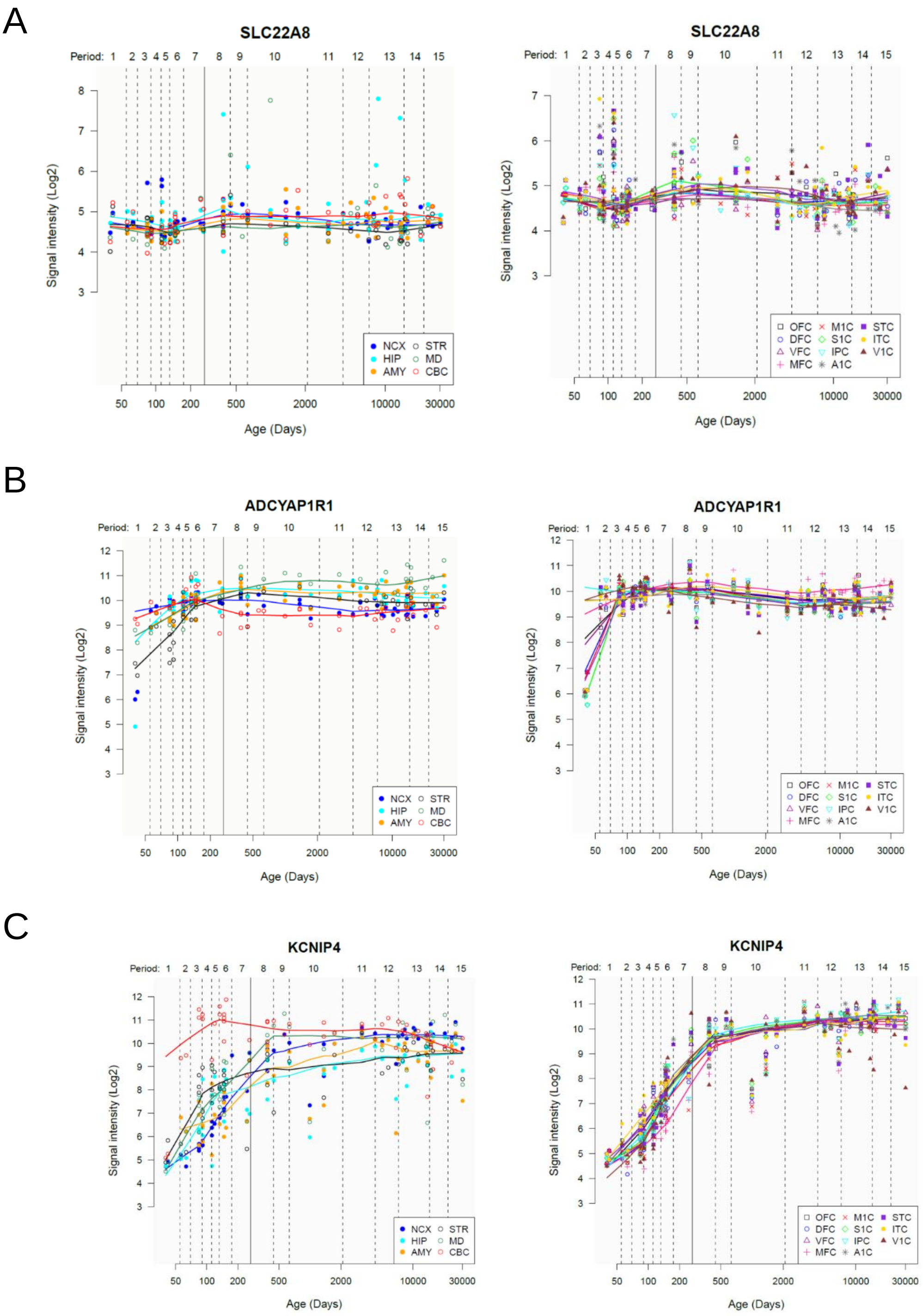

Figure S3. The spatiotemporal expression pattern of *SMAD9*, *NAP1L4*, *ERBB4* in human brain tissues.

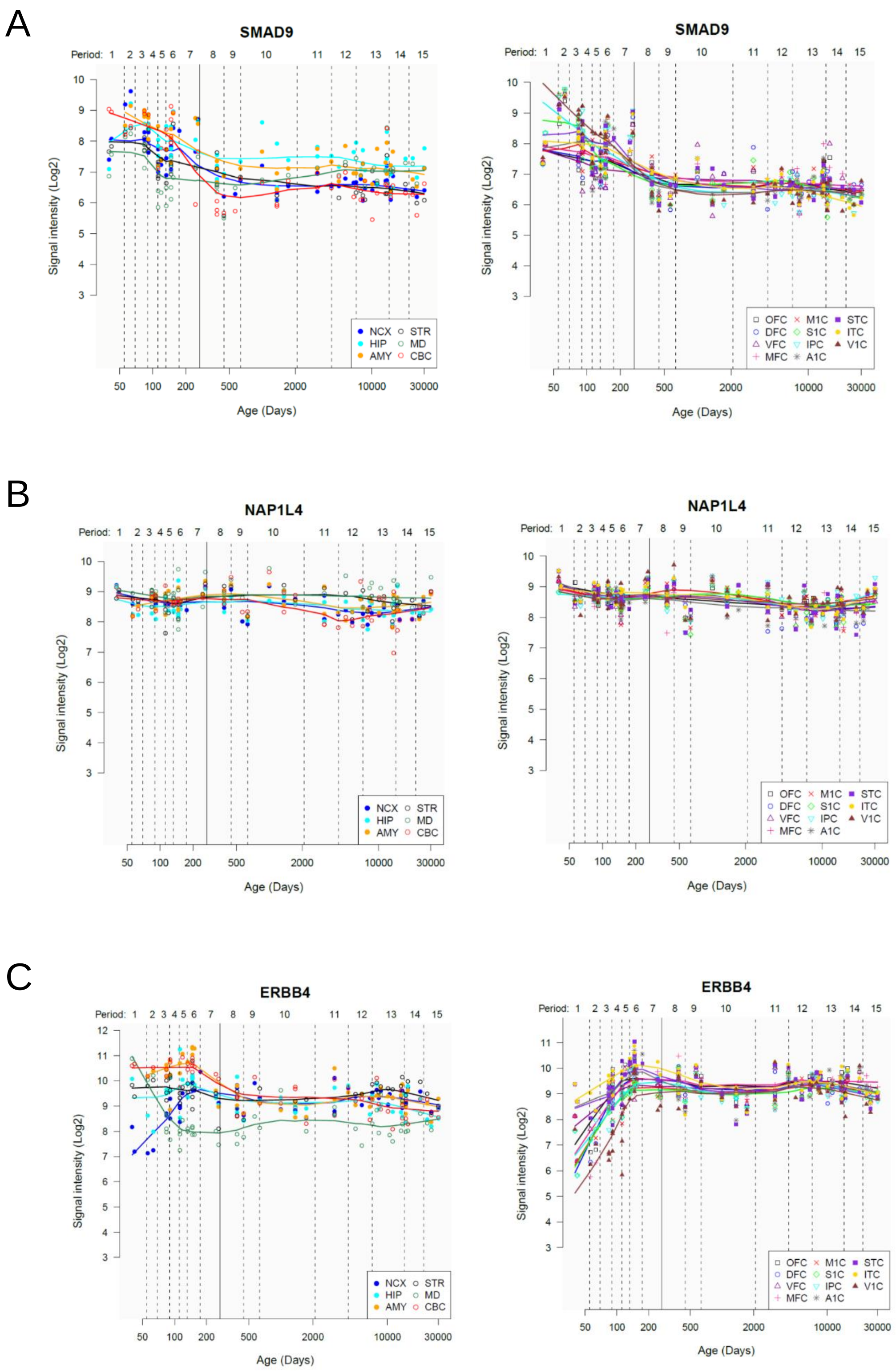

Figure S4. Bulk tissue gene expression of *SLC22A8*, *ADCYAP1R*, *KCNIP4* in human tissues. The bulk tissue gene expressions of candidate genes were analysed on the GTEx website (https://gtexportal.org/home/gene/).

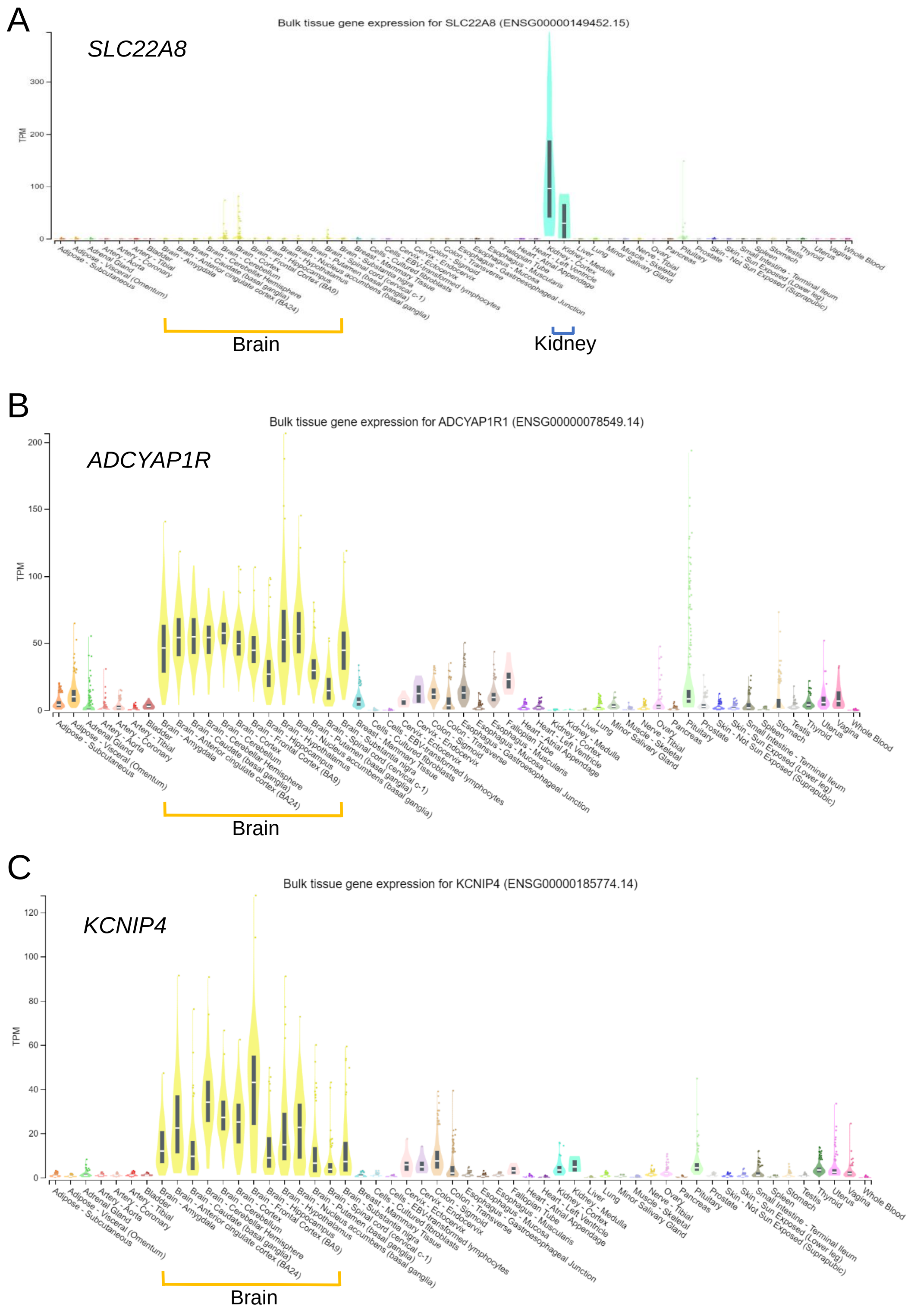

Figure S5. Bulk tissue gene expression of *SMAD9*, *NAP1L4*, *ERBB4* in human tissues.

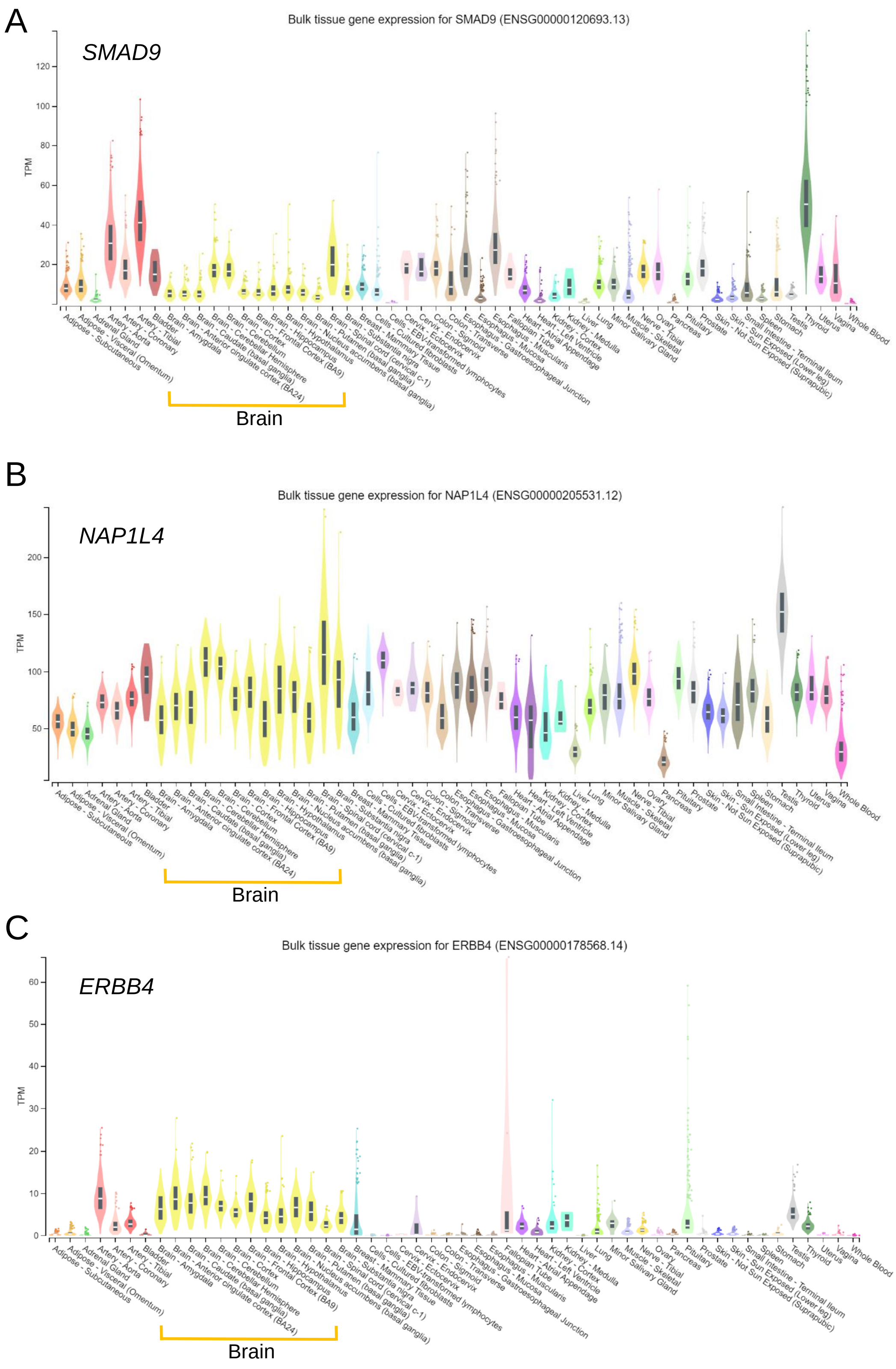

Figure S6. Genome-wide eQTL summary statistics of *SLC22A8*, *ADCYAP1R*, *KCNIP4* in human. The results of candidate genes were obtained from QTLbase (http://www.mulinlab.org/qtlbase).

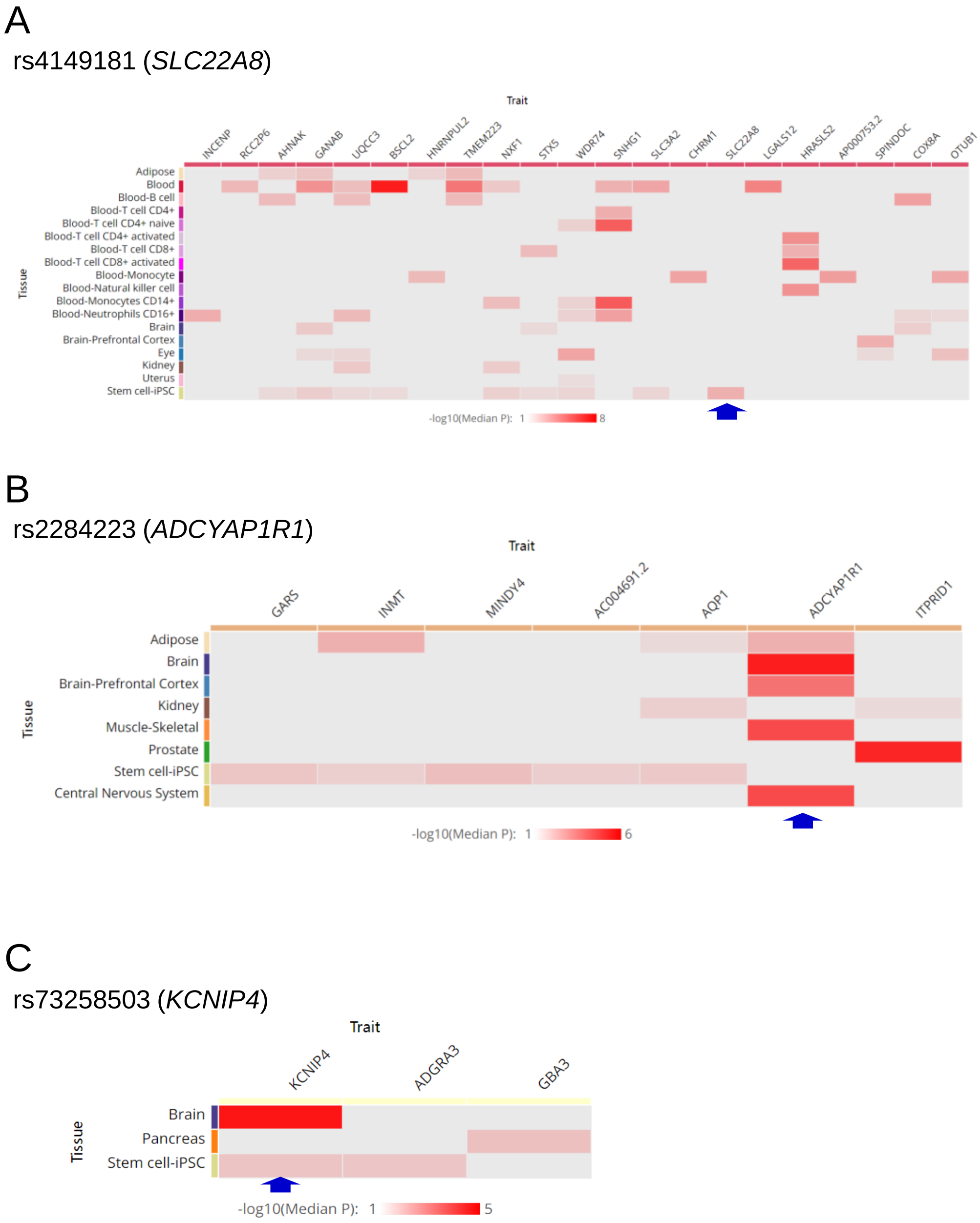

Figure S7. Genome-wide eQTL summary statistics of *SMAD9*, *NAP1L4*, *ERBB4* in human.

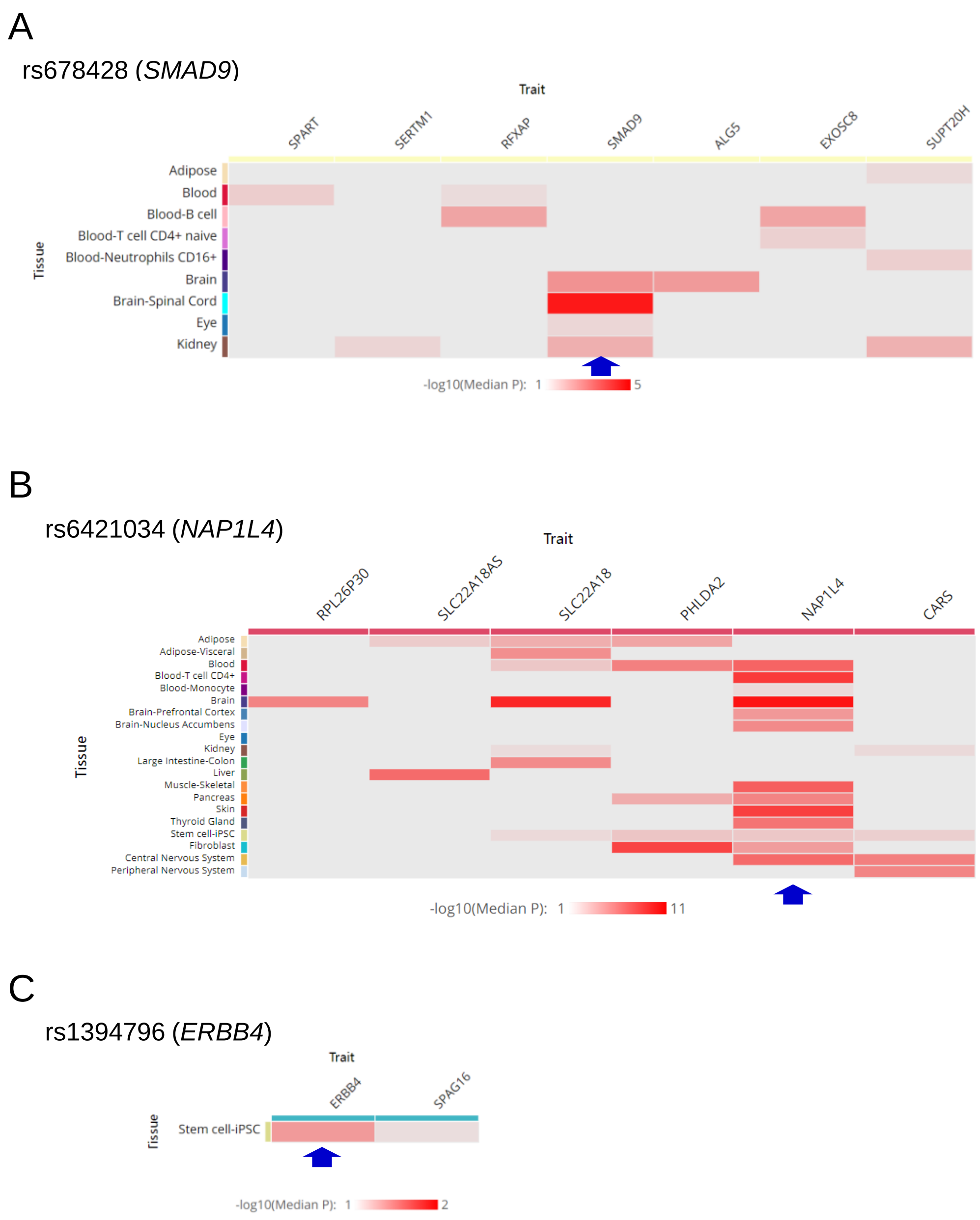

Figure S8. The predictive efficacy of 5 risk gene loci for adverse reactions was verified in CAPEC samples.

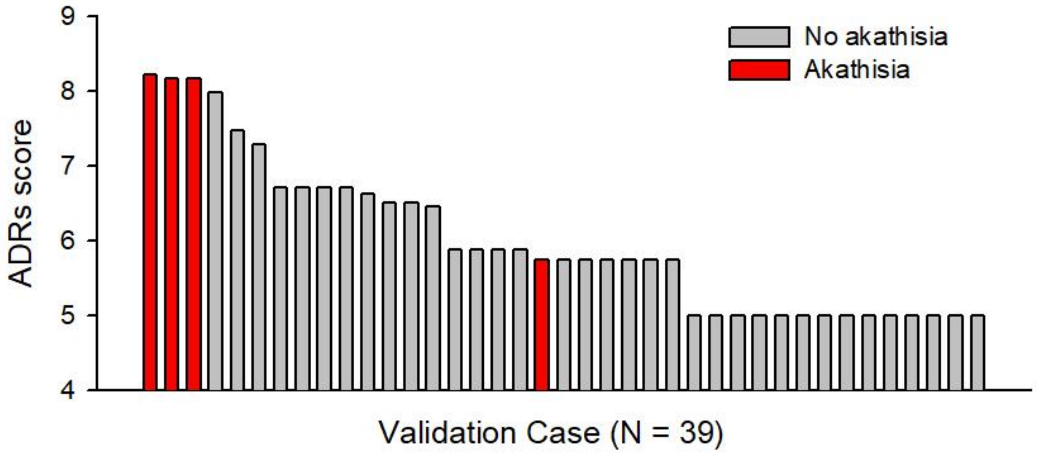

Figure S9. Protein interaction analysis of genes that show genome-wide significance less than $1\times10^{-5}$. The different colored lines indicate the source of evidence, as purple line - experimental evidence, yellow line - textmining evidence, and black line - coexpression evidence. The protein interaction analysis of candidate genes was performed on the STRING website, (https://string-db.org/).

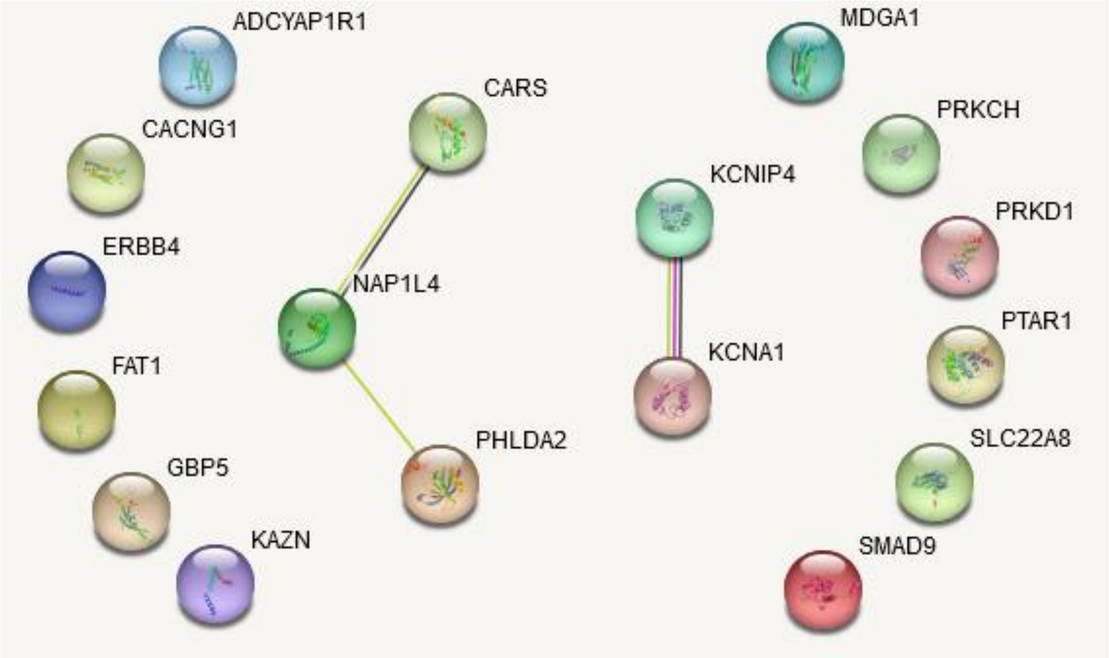

## SUPPLEMENTARY TABLES

Table S1. Genome-wide association results of candidate loci and ADRs without gene annotation.

| CHR | SNP | Position | Minor allele | Major allele | Frequence of minor allele | Functional annotation | Gene | BETA | SE | p value |
|---|---|---|---|---|---|---|---|---|---|---|
| 4 | rs146319527 | 22000008 | A | G | 0.1603 | NA | RP11-17E2.2 | 2.607 | 0.5728 | 5.23E-07 |
| 10 | rs4747269 | 16363505 | C | T | 0.05688 | NA | 26 kb 3' of RP11-461K13.1 | 4.169 | 0.7789 | 1.54E-07 |
| 16 | rs238842 | 18105520 | T | C | 0.2701 | NA | CTA-481E9.4 | 2.182 | 0.4081 | 1.58E-07 |
| 22 | rs9605090 | 20214783 | A | G | 0.08594 | NA | 14 kb 3' of RTN4R | 3.228 | 0.6333 | 5.51E-07 |

Table S2. Association results of genome-wide associated genes with aripiprazole treatment adverse reaction in our study. The SNPs with significance less than $1\times10^{-5}$ are listed, and a total of 120 SNPs that are located in the intron of KCNIP4 are not shown due to their large amount.

| CHR | SNP | BP | Nearby Gene | A1 | A2 | FRQ | BETA | SE | *P* |
|---|---|---|---|---|---|---|---|---|---|
| 1 | rs59572147 | 15256352 | KAZN | C | G | 0.06596 | 3.252 | 0.7029 | 5.17E-06 |
| 1 | rs3806339 | 89735135 | GBP5 | A | G | 0.09115 | 2.885 | 0.6406 | 8.97E-06 |
| 1 | rs138182193 | 89738401 | GBP5 | C | T | 0.09031 | 2.96 | 0.645 | 6.08E-06 |
| 2 | rs10174236 | 213223384 | ERBB4 | T | A | 0.06955 | 3.533 | 0.7581 | 4.42E-06 |
| 2 | rs13383978 | 213223971 | ERBB4 | G | T | 0.06693 | 3.712 | 0.7655 | 1.83E-06 |
| 2 | rs13395813 | 213223983 | ERBB4 | C | A | 0.06693 | 3.712 | 0.7655 | 1.83E-06 |
| 2 | rs10497972 | 213225296 | ERBB4 | C | G | 0.06771 | 3.539 | 0.7606 | 4.55E-06 |
| 2 | rs28623294 | 213225425 | ERBB4 | C | G | 0.06937 | 3.538 | 0.7552 | 3.92E-06 |
| 2 | rs77945488 | 213225704 | ERBB4 | G | A | 0.06771 | 3.539 | 0.7606 | 4.55E-06 |
| 2 | rs11892564 | 213226544 | ERBB4 | G | A | 0.07031 | 3.464 | 0.7493 | 5.23E-06 |
| 2 | rs13384603 | 213227710 | ERBB4 | T | C | 0.0705 | 3.453 | 0.7503 | 5.72E-06 |
| 2 | rs1394799 | 213230879 | ERBB4 | G | A | 0.06789 | 3.529 | 0.7617 | 4.98E-06 |
| 2 | rs6435700 | 213236480 | ERBB4 | A | G | 0.06789 | 3.529 | 0.7617 | 4.98E-06 |
| 2 | rs62184101 | 213251204 | ERBB4 | T | C | 0.06675 | 3.482 | 0.7667 | 7.57E-06 |
| 2 | rs13383860 | 213253930 | ERBB4 | C | T | 0.06955 | 3.401 | 0.7562 | 9.21E-06 |
| 2 | rs62186269 | 213260439 | ERBB4 | A | G | 0.06764 | 3.501 | 0.767 | 6.86E-06 |
| 2 | rs112271765 | 213260485 | ERBB4 | G | A | 0.06764 | 3.501 | 0.767 | 6.86E-06 |
| 2 | rs76907545 | 213262350 | ERBB4 | G | A | 0.06746 | 3.509 | 0.7655 | 6.29E-06 |
| 2 | rs138134225 | 213269660 | ERBB4 | G | T | 0.06136 | 3.863 | 0.7914 | 1.57E-06 |
| 2 | rs1394796 | 213270427 | ERBB4 | T | C | 0.06397 | 3.903 | 0.7796 | 8.60E-07 |
| 2 | rs1394797 | 213270441 | ERBB4 | C | T | 0.0612 | 3.867 | 0.7906 | 1.49E-06 |
| 2 | rs16848495 | 213273682 | ERBB4 | T | C | 0.06005 | 3.992 | 0.7995 | 9.15E-07 |
| 2 | rs80019215 | 213274958 | ERBB4 | T | A | 0.06005 | 3.992 | 0.7995 | 9.15E-07 |
| 2 | rs16848505 | 213275991 | ERBB4 | G | C | 0.06005 | 3.992 | 0.7995 | 9.15E-07 |
| 2 | rs73062317 | 213276062 | ERBB4 | G | A | 0.06005 | 3.992 | 0.7995 | 9.15E-07 |
| 2 | rs16848550 | 213298022 | ERBB4 | T | C | 0.05381 | 3.827 | 0.8441 | 7.83E-06 |
| 2 | rs16848553 | 213299544 | ERBB4 | T | G | 0.05172 | 4.197 | 0.8577 | 1.49E-06 |
| 2 | rs16848556 | 213300321 | ERBB4 | G | A | 0.05438 | 3.833 | 0.8444 | 7.68E-06 |
| 2 | rs16848564 | 213301267 | ERBB4 | G | A | 0.05526 | 3.763 | 0.8373 | 9.36E-06 |
| 2 | rs16848571 | 213302626 | ERBB4 | A | G | 0.05526 | 3.763 | 0.8373 | 9.36E-06 |
| 2 | rs74434401 | 213302777 | ERBB4 | T | G | 0.05526 | 3.763 | 0.8373 | 9.36E-06 |
| 4 | rs62342737 | 187654878 | 7kb 5' of FAT1 | G | A | 0.05078 | 3.742 | 0.8155 | 6.11E-06 |
| 6 | rs804859 | 37614462 | MDGA1 | T | C | 0.1653 | 2.386 | 0.5132 | 4.69E-06 |
| 7 | rs758997 | 31111330 | ADCYAP1R1 | A | G | 0.2917 | 1.958 | 0.4195 | 4.27E-06 |
| 7 | rs2284223 | 31111441 | ADCYAP1R1 | C | T | 0.2947 | 2.166 | 0.3982 | 9.76E-08 |

| | | | | | | | | | |
|---|---|---|---|---|---|---|---|---|---|
| 7 | rs4723044 | 31111708 | ADCYAP1R1 | C | T | 0.2911 | 1.917 | 0.4207 | 7.08E-06 |
| 9 | rs11139962 | 72334016 | PTAR1 | A | G | 0.4582 | -1.703 | 0.3775 | 8.67E-06 |
| 9 | rs11139992 | 72337691 | PTAR1 | T | C | 0.4407 | -1.771 | 0.3926 | 8.80E-06 |
| 9 | rs10867996 | 72348308 | PTAR1 | T | C | 0.458 | -1.698 | 0.3783 | 9.64E-06 |
| 9 | rs1854685 | 72351429 | PTAR1 | T | C | 0.4489 | -1.846 | 0.3872 | 2.71E-06 |
| 9 | rs11140074 | 72355100 | PTAR1 | C | T | 0.458 | -1.698 | 0.3783 | 9.64E-06 |
| 11 | rs12361922 | 2955668 | 5kb 5' of PHLDA2 | C | A | 0.1138 | 2.791 | 0.5718 | 1.58E-06 |
| 11 | rs11024646 | 2955929 | 5.2kb 5' of PHLDA2 | T | C | 0.1138 | 2.791 | 0.5718 | 1.58E-06 |
| 11 | rs12363207 | 2955996 | 5.3kb 5' of PHLDA2 | A | G | 0.1138 | 2.791 | 0.5718 | 1.58E-06 |
| 11 | rs1519 | 2967167 | NAP1L4 | C | G | 0.1185 | 2.674 | 0.5676 | 3.48E-06 |
| 11 | rs3213614 | 2973740 | NAP1L4 | A | G | 0.1185 | 2.674 | 0.5676 | 3.48E-06 |
| 11 | rs4758576 | 2973880 | NAP1L4 | G | A | 0.1185 | 2.674 | 0.5676 | 3.48E-06 |
| 11 | rs4758590 | 2978860 | NAP1L4 | G | A | 0.1185 | 2.674 | 0.5676 | 3.48E-06 |
| 11 | rs7940694 | 2979798 | NAP1L4 | G | T | 0.1185 | 2.674 | 0.5676 | 3.48E-06 |
| 11 | rs737872 | 2983494 | NAP1L4 | G | T | 0.1185 | 2.674 | 0.5676 | 3.48E-06 |
| 11 | rs4758501 | 2990693 | NAP1L4 | G | T | 0.1185 | 2.674 | 0.5676 | 3.48E-06 |
| 11 | rs7946134 | 2996689 | NAP1L4 | A | G | 0.1185 | 2.674 | 0.5676 | 3.48E-06 |
| 11 | rs4758504 | 3000179 | NAP1L4 | A | G | 0.1185 | 2.674 | 0.5676 | 3.48E-06 |
| 11 | rs6578306 | 3002332 | NAP1L4 | C | G | 0.1175 | 2.689 | 0.5697 | 3.35E-06 |
| 11 | rs7113131 | 3003486 | NAP1L4 | C | T | 0.1175 | 2.689 | 0.5697 | 3.35E-06 |
| 11 | rs6578308 | 3007482 | NAP1L4 | A | G | 0.1181 | 2.7 | 0.5704 | 3.15E-06 |
| 11 | rs6421034 | 3007647 | NAP1L4 | C | T | 0.122 | 2.769 | 0.5478 | 6.80E-07 |
| 11 | rs4758622 | 3009921 | NAP1L4 | A | G | 0.1184 | 2.701 | 0.5713 | 3.25E-06 |
| 11 | rs7103117 | 3037732 | CARS | C | T | 0.1359 | 2.489 | 0.5523 | 8.86E-06 |
| 11 | rs4149181 | 62781921 | SLC22A8 | G | A | 0.05339 | 4.436 | 0.7767 | 2.28E-08 |
| 11 | rs4149180 | 62782278 | SLC22A8 | T | C | 0.05339 | 4.436 | 0.7767 | 2.28E-08 |
| 12 | rs41482147 | 5027173 | KCNA1 | A | G | 0.1016 | 2.875 | 0.5925 | 1.80E-06 |
| 12 | rs6489586 | 5029011 | KCNA1 | A | G | 0.1047 | 2.785 | 0.5833 | 2.60E-06 |
| 12 | rs57468930 | 5035398 | KCNA1 | T | C | 0.09661 | 2.948 | 0.6038 | 1.56E-06 |
| 12 | rs17784458 | 5038352 | KCNA1 | T | C | 0.06971 | 3.216 | 0.7062 | 7.23E-06 |
| 13 | rs678428 | 37484026 | SMAD9 | G | A | 0.05469 | 4.077 | 0.7949 | 4.70E-07 |
| 13 | rs660122 | 37490223 | SMAD9 | C | T | 0.05469 | 4.077 | 0.7949 | 4.70E-07 |
| 14 | rs28495988 | 30371420 | PRKD1 | G | A | 0.07743 | 3.249 | 0.6766 | 2.29E-06 |
| 14 | rs56132967 | 61644977 | 9.3kb 5' of PRKCH | T | G | 0.09974 | 2.774 | 0.61 | 7.38E-06 |
| 17 | rs11651301 | 65045326 | CACNG1 | A | G | 0.2279 | 2.003 | 0.4357 | 5.86E-06 |
| 17 | rs1799938 | 65052304 | CACNG1 | A | G | 0.08203 | 2.889 | 0.6388 | 8.21E-06 |

Table S3. The GO terms associated with adverse effects of 6-week aripiprazole treatment with candidate genes that show the genome-wide significance level less than $1\times10^{-5}$.

| ID | Description | p value | p adjust | Gene ID* |
|---|---|---|---|---|
| GO:0004697 | protein kinase C activity | 8.49095E-05 | 0.002887 | PRKCH/PRKD1 |
| GO:0004698 | calcium-dependent protein kinase C activity | 8.49095E-05 | 0.002887 | PRKCH/PRKD1 |
| GO:0009931 | calcium-dependent protein serine/threonine kinase activity | 0.000178381 | 0.003306 | PRKCH/PRKD1 |
| GO:0010857 | calcium-dependent protein kinase activity | 0.000194499 | 0.003306 | PRKCH/PRKD1 |
| GO:0010959 | regulation of metal ion transport | 0.000284853 | 0.142141 | ADCYAP1R1/CACNG1/KCNA1/KCNIP4 |
| GO:0005244 | voltage-gated ion channel activity | 0.000615392 | 0.006974 | CACNG1/KCNA1/KCNIP4 |
| GO:0022832 | voltage-gated channel activity | 0.000615392 | 0.006974 | CACNG1/KCNA1/KCNIP4 |
| GO:0035637 | multicellular organismal signaling | 0.000646924 | 0.148876 | CACNG1/KCNA1/KCNIP4 |
| GO:0034703 | cation channel complex | 0.000714863 | 0.021611 | CACNG1/KCNA1/KCNIP4 |
| GO:0098982 | GABA-ergic synapse | 0.001637381 | 0.021611 | ERBB4/MDGA1 |
| GO:0099056 | integral component of presynaptic membrane | 0.001637381 | 0.021611 | ERBB4/KCNA1 |
| GO:0034702 | ion channel complex | 0.001708777 | 0.021611 | CACNG1/KCNA1/KCNIP4 |
| GO:0098889 | intrinsic component of presynaptic membrane | 0.002054114 | 0.021611 | ERBB4/KCNA1 |
| GO:1902495 | transmembrane transporter complex | 0.002111354 | 0.021611 | CACNG1/KCNA1/KCNIP4 |
| GO:1990351 | transporter complex | 0.00226411 | 0.021611 | CACNG1/KCNA1/KCNIP4 |
| GO:0008076 | voltage-gated potassium channel complex | 0.002305153 | 0.021611 | KCNA1/KCNIP4 |
| GO:1901379 | regulation of potassium ion transmembrane transport | 0.002642523 | 0.148876 | KCNA1/KCNIP4 |
| GO:0034705 | potassium channel complex | 0.002735505 | 0.021905 | KCNA1/KCNIP4 |
| GO:0005261 | cation channel activity | 0.002805701 | 0.02446 | CACNG1/KCNA1/KCNIP4 |
| GO:0022836 | gated channel activity | 0.002877615 | 0.02446 | CACNG1/KCNA1/KCNIP4 |
| GO:0016324 | apical plasma membrane | 0.002920716 | 0.021905 | FAT1/KCNA1/SLC22A8 |
| GO:1904062 | regulation of cation transmembrane transport | 0.00293878 | 0.148876 | CACNG1/KCNA1/KCNIP4 |
| GO:0043266 | regulation of potassium ion transport | 0.003630724 | 0.148876 | KCNA1/KCNIP4 |

| | | | | |
|---|---|---|---|---|
| GO:0099055 | integral component of postsynaptic membrane | 0.004030352 | 0.027333 | ERBB4/KCNA1 |
| GO:0098936 | intrinsic component of postsynaptic membrane | 0.004373312 | 0.027333 | ERBB4/KCNA1 |
| GO:0099106 | ion channel regulator activity | 0.004714665 | 0.031871 | CACNG1/KCNIP4 |
| GO:0005267 | potassium channel activity | 0.004792143 | 0.031871 | KCNA1/KCNIP4 |
| GO:0045177 | apical part of cell | 0.00486858 | 0.028088 | FAT1/KCNA1/SLC22A8 |
| GO:0034329 | cell junction assembly | 0.005417029 | 0.148876 | ERBB4/MDGA1/PRKCH |
| GO:0046873 | metal ion transmembrane transporter activity | 0.005626548 | 0.031871 | CACNG1/KCNA1/KCNIP4 |
| GO:0005216 | ion channel activity | 0.005736796 | 0.031871 | CACNG1/KCNA1/KCNIP4 |
| GO:0022843 | voltage-gated cation channel activity | 0.006464556 | 0.031871 | CACNG1/KCNA1 |
| GO:0010675 | regulation of cellular carbohydrate metabolic process | 0.006824895 | 0.148876 | ADCYAP1R1/PHLDA2 |
| GO:0061337 | cardiac conduction | 0.006824895 | 0.148876 | CACNG1/KCNIP4 |
| GO:0099699 | integral component of synaptic membrane | 0.006874853 | 0.03304 | ERBB4/KCNA1 |
| GO:0005911 | cell-cell junction | 0.006979375 | 0.03304 | ADCYAP1R1/FAT1/KAZN |
| GO:0042734 | presynaptic membrane | 0.007048462 | 0.03304 | ERBB4/KCNA1 |
| GO:0016247 | channel regulator activity | 0.007198024 | 0.031871 | CACNG1/KCNIP4 |
| GO:0034765 | regulation of ion transmembrane transport | 0.007536561 | 0.148876 | CACNG1/KCNA1/KCNIP4 |
| GO:0015267 | channel activity | 0.00768134 | 0.031871 | CACNG1/KCNA1/KCNIP4 |
| GO:0022803 | passive transmembrane transporter activity | 0.007725531 | 0.031871 | CACNG1/KCNA1/KCNIP4 |
| GO:0051092 | positive regulation of NF-kappaB transcription factor activity | 0.00783818 | 0.148876 | PRKCH/PRKD1 |
| GO:0099240 | intrinsic component of synaptic membrane | 0.007946289 | 0.033967 | ERBB4/KCNA1 |
| GO:0015079 | potassium ion transmembrane transporter activity | 0.007967632 | 0.031871 | KCNA1/KCNIP4 |
| GO:0044224 | juxtaparanode region of axon | 0.008152198 | 0.033967 | KCNA1 |
| GO:0001764 | neuron migration | 0.008319971 | 0.148876 | ERBB4/MDGA1 |
| GO:0018342 | protein prenylation | 0.00845058 | 0.148876 | PTAR1 |
| GO:0034350 | regulation of glial cell apoptotic process | 0.00845058 | 0.148876 | PRKCH |
| GO:0050861 | positive regulation of B cell receptor signaling pathway | 0.00845058 | 0.148876 | PRKCH |
| GO:0097354 | prenylation | 0.00845058 | 0.148876 | PTAR1 |

| | | | | |
|---|---|---|---|---|
| GO:1901725 | regulation of histone deacetylase activity | 0.00845058 | 0.148876 | PRKD1 |
| GO:0051260 | protein homooligomerization | 0.009738374 | 0.148876 | GBP5/KCNA1 |
| GO:0033270 | paranode region of axon | 0.009775143 | 0.036657 | KCNA1 |
| GO:1990454 | L-type voltage-gated calcium channel complex | 0.009775143 | 0.036657 | CACNG1 |

* Gene ID includes the candidate genes with genome-wide significance less than $1\times10^{-5}$. This table present the Gene Ontology (GO) term with $p < 0.001$.
